# Association of Air Pollution Exposure With Psychotic Experiences During Adolescence

**DOI:** 10.1001/jamapsychiatry.2019.0056

**Published:** 2019-03-27

**Authors:** Joanne B. Newbury, Louise Arseneault, Sean Beevers, Nutthida Kitwiroon, Susanna Roberts, Carmine M. Pariante, Frank J. Kelly, Helen L. Fisher

**Affiliations:** 1 King’s College London, Social, Genetic & Developmental Psychiatry Centre, Institute of Psychiatry, Psychology & Neuroscience, London, United Kingdom; 2 King’s College London, Environmental Research Group, MRC-PHE (Medical Research Council–Public Health England) Centre for Environment and Health, London, United Kingdom; 3 King’s College London, Department of Psychology, Institute of Psychiatry, Psychology and Neuroscience, London, United Kingdom; 4 King’s College London, Department of Psychological Medicine, Institute of Psychiatry, Psychology and Neuroscience, London, United Kingdom

## Abstract

**Importance:**

Urbanicity is a well-established risk factor for clinical (eg, schizophrenia) and subclinical (eg, hearing voices and paranoia) expressions of psychosis. To our knowledge, no studies have examined the association of air pollution with adolescent psychotic experiences, despite air pollution being a major environmental problem in cities.

**Objectives:**

To examine the association between exposure to air pollution and adolescent psychotic experiences and test whether exposure mediates the association between urban residency and adolescent psychotic experiences.

**Design, Setting, and Participants:**

The Environmental-Risk Longitudinal Twin Study is a population-based cohort study of 2232 children born during the period from January 1, 1994, through December 4, 1995, in England and Wales and followed up from birth through 18 years of age. The cohort represents the geographic and socioeconomic composition of UK households. Of the original cohort, 2066 (92.6%) participated in assessments at 18 years of age, of whom 2063 (99.9%) provided data on psychotic experiences. Generation of the pollution data was completed on October 4, 2017, and data were analyzed from May 4 to November 21, 2018.

**Exposures:**

High-resolution annualized estimates of exposure to 4 air pollutants—nitrogen dioxide (NO_2_), nitrogen oxides (NO_x_), and particulate matter with aerodynamic diameters of less than 2.5 (PM_2.5_) and less than 10 μm (PM_10_)—were modeled for 2012 and linked to the home addresses of the sample plus 2 commonly visited locations when the participants were 18 years old.

**Main Outcomes and Measures:**

At 18 years of age, participants were privately interviewed regarding adolescent psychotic experiences. Urbanicity was estimated using 2011 census data.

**Results:**

Among the 2063 participants who provided data on psychotic experiences, sex was evenly distributed (52.5% female). Six hundred twenty-three participants (30.2%) had at least 1 psychotic experience from 12 to 18 years of age. Psychotic experiences were significantly more common among adolescents with the highest (top quartile) level of annual exposure to NO_2_ (odds ratio [OR], 1.71; 95% CI, 1.28-2.28), NO_x_ (OR, 1.72; 95% CI, 1.30-2.29), and PM_2.5_ (OR, 1.45; 95% CI, 1.11-1.90). Together NO_2_ and NO_x_ statistically explained 60% of the association between urbanicity and adolescent psychotic experiences. No evidence of confounding by family socioeconomic status, family psychiatric history, maternal psychosis, childhood psychotic symptoms, adolescent smoking and substance dependence, or neighborhood socioeconomic status, crime, and social conditions occurred.

**Conclusions and Relevance:**

In this study, air pollution exposure—particularly NO_2_ and NO_x_—was associated with increased odds of adolescent psychotic experiences, which partly explained the association between urban residency and adolescent psychotic experiences. Biological (eg, neuroinflammation) and psychosocial (eg, stress) mechanisms are plausible.

## Key Points

**Question:**

Is exposure to air pollution associated with adolescent psychotic experiences?

**Findings:**

In this nationally representative cohort study of 2232 UK-born children, significant associations were found between outdoor exposure to nitrogen dioxide, nitrogen oxides, and particulate matter and reports of psychotic experiences during adolescence. Moreover, nitrogen dioxide and nitrogen oxides together explained 60% of the association between urban residency and adolescent psychotic experiences.

**Meaning:**

The association between urban residency and adolescent psychotic experiences is partly explained by the higher levels of outdoor air pollution in urban settings.

## Teaser

This population-based cohort study examines the association between exposure to air pollution and adolescent psychotic experiences and whether exposure explains the association between urbanicity and psychosis in a representative sample of UK adolescents.

## Introduction

Several decades have passed since Faris and Dunham^1^ first documented higher rates of schizophrenia in inner-city Chicago relative to the city outskirts. The body of research since their ecological study suggests that urban upbringing is associated with a 2-fold adulthood risk for psychotic disorder.^2^ Given that 70% of the world’s population will be urban by 2050,^3^ uncovering the mechanisms linking the urban environment to psychosis and developing preventive interventions constitute an urgent health priority.

Epidemiological research to date has mostly examined adverse social features in the urban environment, such as neighborhood deprivation and crime.^4,5,6,7^ However, a key feature of the urban environment remains underresearched. Air pollution is a major worldwide health issue,^8^ particularly in cities, where pollution levels frequently exceed limits set by the World Health Organization (WHO)^9^ and the European Union.^10^ Primary air pollutants are typically released through combustion processes; principal sources include road transport, industry, and domestic activity.^11,12^ In addition, some air pollutants are secondarily formed in the atmosphere through a series of photochemical reactions. Pollution has long been implicated in a range of physical health problems, including cardiovascular and respiratory disease.^13,14^ Growing evidence now links air pollution to psychiatric disorders. Associations have been documented between air pollution and anxiety,^15^ depression,^16^ autism spectrum disorder,^17^ and Alzheimer-like disease.^18^ A handful of studies have also examined associations between air pollution and adult psychotic disorders,^19,20,21^ but findings are inconsistent.

Moreover, few studies have used high-resolution measures of air pollution to examine associations with psychosis, and none have examined associations with adolescent psychotic experiences, such as hearing voices and extreme paranoia. These early psychotic phenomena are a developmental risk factor for adult psychotic disorder^22,23^ and other serious mental health problems^24^ and are thought to lie on an etiological continuum with clinical psychosis.^25^ Focusing on adolescent psychotic experiences (vs adult disorders) provides several analytic advantages. First, infants and youth are most vulnerable to air pollution owing to the juvenility of the brain and respiratory system.^26^ Second, early psychotic phenomena are recognized as an important target for early intervention.^27^ Third, psychotic phenomena are also approximately twice as common among youth raised in cities.^28,29,30,31,32^ Air pollution is a plausible component of this association. Finally, subclinical psychotic experiences are relatively common among children and adolescents,^33^ thereby increasing our power to detect associations in the general population.

The present study uses data from a nationally representative cohort of 2232 children, interviewed repeatedly from birth to 18 years of age. A battery of phenotypic, family-, and neighborhood-level measures has been collected for 2 decades. High-resolution estimates of levels of 4 ambient (outdoor) air pollutants, including nitrogen dioxide (NO_2_), nitrogen oxides (NO_x_), and particulate matter with aerodynamic diameter of less than 2.5 (PM_2.5_) and less than 10 μm (PM_10_), have been linked to the addresses of the sample in 2012, the year before the interviews at 18 years of age. We also incorporated pollution data for 2 additional addresses where adolescents spent their time to create a more comprehensive picture of ambient pollution exposure. Using these data, we tested the hypotheses that (1) psychotic experiences are more common among adolescents exposed to higher levels of air pollution, and (2) levels of air pollution partly explain the association between urbanicity and adolescent psychotic experiences. Analyses controlled for a range of potential individual-, family-, and neighborhood-level confounders.

## Methods

### Study Cohort

Participants were members of the Environmental Risk (E-Risk) Longitudinal Twin Study, which tracks the development of a nationally representative birth cohort of 2232 twin children born from January 1, 1994, through December 4, 1995, across England and Wales and initially assessed at 5 years of age. This sample included 1242 (55.6%) monozygotic and 990 (44.4%) dizygotic twin pairs; sex was evenly distributed within zygosity (1092 male [48.9%]). Follow-up home visits were conducted when participants were aged 7 (98% participation), 10 (96% participation), 12 (96% participation), and 18 (93% participation) years. At 18 years of age, the E-Risk sample included 2066 participants. No differences occurred between those who did and did not participate at 18 years of age in terms of socioeconomic status (χ^2^ = 0.86; *P* = .65), IQ scores (2-tailed independent *t* = 0.98; *P* = .33), or internalizing (2-tailed independent *t* = 0.40; *P* = .69) or externalizing (2-tailed independent *t* = 0.41; *P* = .68) behavior problems at 5 years of age. E-Risk participants are representative of UK households across the spectrum of neighborhood socioeconomic conditions: at 18 years of age, 27.0% of E-Risk participants (n = 489) lived in wealthy-achiever neighborhoods compared with 25.4% of households nationwide; 7.2% (n= 131) vs 11.5% lived in urban-prosperity neighborhoods; 26.8% (n = 484) vs 27.4% lived in comfortably off neighborhoods; 13.2% (n = 239) vs 13.8% lived in moderate-means neighborhoods; and 25.8% (n = 468) vs 21.2% lived in hard-pressed neighborhoods.^34^ Most of the 2066 participants (1475 [71.4%]) lived at the same address from 12 to 18 years of age. The joint South London and Maudsley and the Institute of Psychiatry research ethics committee approved each phase of the study. Parents gave written informed consent, and participants gave written assent at 5 to 12 years of age and written informed consent at 18 years of age. Table 1 displays sociodemographic characteristics of the E-Risk participants at 18 years of age. Further details about the sample are reported elsewhere,^35^ and in the eMethods in the Supplement.

**Table 1.** Sociodemographic Characteristics of the E-Risk Longitudinal Twin Study Participants at 18 Years of Age

Variable	No. (%) of Participants			χ^2 ^Test^a^	*P* Value
	All	Adolescent Psychotic Experiences	No Adolescent Psychotic Experiences		
Total	2063 (100)	623 (30.2)	1440 (69.8)	NA	NA
Sex					
Male	980 (47.5)	305 (31.1)	675 (68.9)	0.8	.39
Female	1083 (52.5)	318 (29.4)	765 (70.6)		
Zygosity					
MZ	1164 (56.4)	346 (29.7)	818 (70.3)	0.3	.59
DZ	899 (43.6)	277 (30.8)	622 (69.2)		
Family SES					
Low	691 (33.5)	255 (36.9)	436 (63.1)	32.1	<.001
Middle	683 (33.1)	210 (30.7)	473 (69.3)		
High	689 (33.4)	158 (22.9)	531 (77.1)		
Neighborhood SES					
Hard pressed	468 (25.8)	160 (34.2)	308 (65.8)	24.5	<.001
Moderate means	239 (13.2)	83 (34.7)	156 (65.3)		
Comfortably off	484 (26.7)	146 (30.2)	338 (69.8)		
Urban prosperity	131 (7.2)	42 (32.1)	89 (67.9)		
Wealthy achievers	489 (27.0)	104 (21.3)	385 (78.7)		
Urbanicity					
Rural	366 (19.7)	82 (22.4)	284 (77.6)	15.9	<.001
Intermediate	897 (48.4)	262 (29.2)	635 (70.8)		
Urban	592 (31.9)	204 (34.5)	388 (65.5)		

Abbreviations: E-Risk, Environmental Risk; SES, socioeconomic status.

^a^ Calculated as test of differences in distribution of psychotic experiences by sociodemographic variables.

### Measures

#### Adolescent Psychotic Experiences

At 18 years of age, each E-Risk participant was privately interviewed by a research worker about 13 psychotic experiences occurring since 12 years of age. Data on psychotic experiences are available for 2063 participants (99.9%) of the sample interviewed. Seven items pertained to delusions and hallucinations,^28^ such as “Have you ever thought you were being watched, followed, or spied on?” and “Do you hear voices that others cannot?” Six items pertained to unusual experiences which drew on item pools since formalized in prodromal psychosis instruments, including the PRIME (Prevention Through Risk Identification, Management, Education) screen and Structured Interview for Prodromal Syndromes,^36^ such as “People or places I know seem different” and “My thinking is unusual or frightening.” Further information on this measure is provided in the eMethods in the Supplement. Research workers coded each item 0 for not present, 1 for probably present, or 2 for definitely present. All 13 items were summed (range, 0-18; mean [SD] score, 1.19 [2.58]), and scores were placed into an ordinal scale. Just more than 30% of participants had at least 1 psychotic experience from 12 to 18 years of age; 1440 (69.8%) reported no psychotic experiences (coded 0); 319 (15.5%) reported 1 or 2 psychotic experiences (coded 1); 166 (8.0%) reported 3 to 5 psychotic experiences (coded 2); and 138 (6.7%) reported 6 or more psychotic experiences (coded 3). This finding is similar to the prevalence of self-reported psychotic experiences in other community samples of teenagers and young adults.^37,38^

#### Adolescent Psychotic Symptoms

Adolescent psychotic symptoms were recorded as responses to the 7 hallucination/delusion items assessed at 18 years of age, verified by health care professionals (eMethods in the Supplement). A conservative approach was taken in designating an adolescent’s report as a symptom. After clinical verification by a team of experts, 59 (2.9%) adolescents reported having at least 1 definite psychotic symptom from 12 to 18 years of age.

#### Ambient Air Pollution

Pollution exposure estimates were modeled for 2012, when participants were 17 years of age, and linked to the latitude-longitude coordinates of participants’ residential addresses at 18 years of age (or where the participant spent most of their time) plus 2 additional addresses where the participants reported spending their time. The most common locations were home, school, work, and shops. Pollution data for the primary addresses were available for 2014 participants (97.5%) (eTable 1 in the Supplement). We decided to model pollution data for 2012 to capitalize on recent developments in pollution models^39^ and create a more comprehensive picture of pollution exposure by incorporating the additional addresses obtained at 18 years of age. Pollution exposure estimates were modeled using the local-scale Community Multiscale Air Quality (CMAQ-urban) Modeling System, which is a coupled regional chemical transport model and street-scale dispersion model. CMAQ-urban uses a new generation of road traffic emissions inventory in the United Kingdom to model air quality down to individual streets, providing hourly estimates of pollutants at 20 × 20-m grid points throughout the United Kingdom (ie, address level). Full details on the creation and validation of this model have been described previously.^40,41^ The pollution estimates achieved good model performance against ground-based measurements (eMethods and eTable 2 in the Supplement). Participants’ exposure to several pollutants was estimated by calculating the mean levels of the pollutant across the year at as many as 3 locations where participants reported spending most of their time, and then calculating the mean across the locations (ie, [annual pollution exposure in location 1 + location 2 + location 3]/3). Pollutants include NO_2_ (regulated gaseous pollutant), NO_x_ (regulated gaseous pollutant, composed of NO_2_ and nitric oxide), and PM_2.5_ and PM_10_ (regulated pollutants composed of inorganic aerosols, carbonaceous aerosols, and dusts). To index the worst levels of air pollution while retaining statistical power and ensuring parity between the measures, air pollutants were dichotomized at the top quartile of exposure in this sample (eMethods in the Supplement provides further detail on the pollution measure and cutoffs). These quartile cutoffs in micrograms per cubic meter were 26.0 μg/m^3^ for NO_2_, 33.0 μg/m^3^ for NO_x_, 12.4 μg/m^3^ for PM_2.5_, and 17.6 μg/m^3^ for PM_10_. All air pollutants were highly correlated (*r* = 0.56-0.97; *P* < .001). We examined the individual associations of each pollutant with adolescent psychotic experiences because pollutants may have differential health effects.

### Other Variables

Urbanicity^42^ was used in mediation models to test whether air pollutants mediated the association between urban residency and adolescent psychotic experiences. A 3-level urbanicity score was derived from classifications from 2011 census data, which combined residential density, output area, and contextual data (592 of 1858 participants with available data [31.9%] lived in the most urban settings at 18 years of age). Analyses controlled for a range of potential covariates that might confound the association^43^ between air pollution and adolescent psychotic experiences, including family socioeconomic status,^44^ family psychiatric history,^45,46^ maternal psychosis,^47,48^ childhood psychotic symptoms,^22,49^ adolescent smoking,^47^ cannabis dependence,^47^ alcohol dependence,^47^ neighborhood socioeconomic status,^50^ neighborhood crime, and neighborhood social conditions.^51,52,53,54,55^ All covariates are described in detail in the eMethods in the Supplement.

### Statistical Analyses

Data were analyzed from May 4 to November 21, 2018. Statistical analyses used Stata software (version 14.1; StataCorp) and followed 3 main steps. First, we used linear regression to check whether urban neighborhoods were more polluted in this cohort. Second, we used ordinal logistic regression (psychotic experiences were placed on an ordinal scale) to test the association of each pollutant with adolescent psychotic experiences. We adjusted in a stepwise manner for potential confounders before controlling for all potential confounders simultaneously. We conducted several sensitivity analyses, using (1) urbanicity as an additional control variable to account comprehensively for urban factors correlated with air pollution; (2) the 71.4% of adolescents who did not move between residences from 12 to 18 years, to keep neighborhood conditions (and therefore air pollution exposure) as consistent over time as possible; (3) pollution variables categorized at different thresholds to check the sensitivity of our top quartile cutoff; (4) adolescent psychotic symptoms as the outcome to check whether associations extended to this clinically verified phenotype; and (5) a 2-pollutant model (NO_x_ and PM_2.5_) to investigate copollutant confounding. Third, we used KHB (Karlson, Holm, and Breen) pathway decomposition^56^ to test whether pollution levels mediated the association between urbanicity and adolescent psychotic experiences, again controlling for potential confounders. The level of statistical significance was set at 2-sided *P* < .05. Because the E-Risk Study uses a twin sample, analyses were adjusted for the nonindependence of twin observations using the CLUSTER command in Stata. This procedure is derived from the Huber-White variance estimator and provides robust standard errors adjusted for within-cluster correlated data.^57^ Given the prevalence of psychotic experiences, odds ratios (ORs) are not a good approximation for risk ratios and should be strictly interpreted as an increase in odds.^58^

## Results

A total of 2063 participants provided data on psychotic experiences at 18 years of age. Of these, 980 (47.5%) were male and 1083 (52.5%) were female. Characteristics of the study group are shown in Table 1.

### Are Urban Neighborhoods More Polluted?

**Figure 1.**
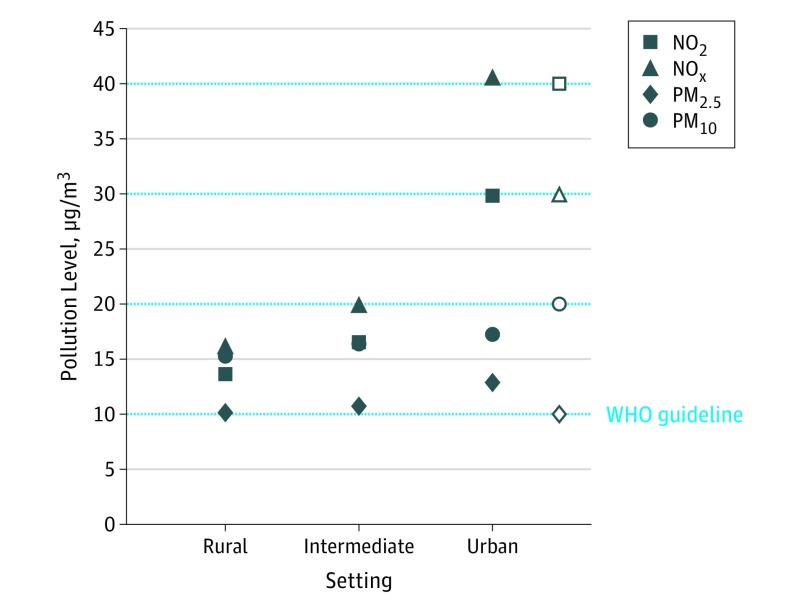
Air Pollution Levels in Rural, Intermediate, and Urban Settings Annualized mean exposure levels of nitrogen dioxide (NO_2_), nitrogen oxides (NO_x_), and particulate matter with aerodynamic diameters of less than 2.5 μm (PM_2.5_) and less than 10 μm (PM_10_) according to level of urbanicity. Clear markers highlight the different World Health Organization (WHO) guidelines for these pollutants.

Figure 1 shows that higher mean levels of NO_2_, NO_x_, PM_2.5_, and PM_10_ were estimated in urban vs rural neighborhoods. Mean levels of NO_x_ (40.6 μm) and PM_2.5_ (12.9 μm) in urban settings exceeded WHO guidelines (40 μm and 10 μm, respectively). Urbanicity was significantly associated with levels of NO_2_ (unstandardized β, 8.68; 95% CI, 8.02-9.35), NO_x_ (unstandardized β, 13.22; 95% CI, 12.03-14.42), PM_2.5_ (unstandardized β, 1.46; 95% CI 1.30-1.63), and PM_10_ (unstandardized β, 0.98; 95% CI, 0.78-1.18). Standardized βs (which may be interpreted as correlations and therefore compared across pollutants) were 0.64 for NO_2_, 0.58 for NO_x_, 0.49 for PM_2.5_, and 0.26 for PM_10_.

### Is Air Pollution Associated With Adolescent Psychotic Experiences?

**Figure 2.**
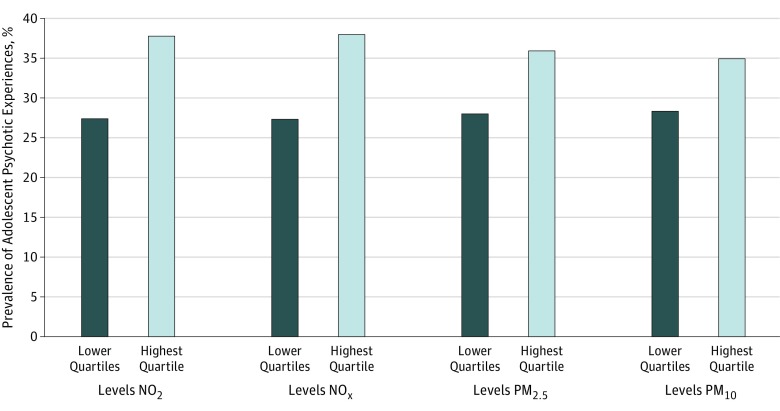
Prevalence of Adolescent Psychotic Experiences According to Exposure to Air Pollutants Air pollutants include nitrogen dioxide (NO_2_), nitrogen oxides (NO_x_), and particulate matter with aerodynamic diameters of less than 2.5 μm (PM_2.5_) and less than 10 μm (PM_10_). Prevalence of adolescent psychotic experiences is split by the highest quartile vs lower quartiles of exposure to each pollutant.

Figure 2 and Table 2 show that adolescents exposed to the highest (top quartile) annual levels of air pollution reported higher rates of psychotic experiences than adolescents exposed to lower levels of pollution. Associations among NO_2_, NO_x_, and PM_2.5_ exposures and adolescent psychotic experiences were slightly attenuated but remained significant after adjusting for family-level factors (model 2), childhood psychotic symptoms (model 3), adolescent substance use (model 4), neighborhood factors (model 5), and after considering all potential confounders simultaneously (model 6). For example, the fully adjusted association between NO_x_ and adolescent psychotic experiences was an OR of 1.72 (95% CI, 1.30-2.29); for NO_2_, an OR of 1.71 (95% CI, 1.28-2.28); and for PM_2.5_, an OR of 1.45 (95% CI, 1.11-1.90). Particulate matter with aerodynamic diameters of less than 10 μm was no longer significantly associated with adolescent psychotic experiences after adjusting for neighborhood factors (OR, 1.24; 95% CI, 0.96-1.61) and all confounders simultaneously (OR, 1.27; 95% CI, 0.98-1.65).

**Table 2.** Association Between Top Quartile of Annualized Mean Levels of Air Pollutants and Adolescent Psychotic Experiences^a^

Model	Pollutants, OR (95% CI)			
	NO_2_	NO*_x_*	PM_2.5_	PM_10_
Model 1 (unadjusted)	1.83 (1.42-2.36)^b^	1.84 (1.43-2.36)^b^	1.58 (1.23-2.03)^b^	1.39 (1.08-1.79)^c^
Model 2 (family factors)^d^	1.83 (1.42-2.37)^b^	1.83 (1.42-2.35)^b^	1.59 (1.23-2.05)^b^	1.39 (1.08-1.79)^c^
Model 3 (childhood psychotic symptoms)	1.84 (1.43-2.37)^b^	1.85 (1.44-2.37)^b^	1.61 (1.26-2.07)^b^	1.37 (1.07-1.77)^c^
Model 4 (adolescent substance use)^e^	1.84 (1.42-2.38)^b^	1.84 (1.43-2.37)^b^	1.55 (1.20-1.98)^f^	1.38 (1.08-1.78)^c^
Model 5 (neighborhood factors)^g^	1.62 (1.22-2.14)^f^	1.63 (1.23-2.15)^f^	1.38 (1.06-1.79)^c^	1.24 (0.96-1.61)^h^
Model 6 (all covariates simultaneously)	1.71 (1.28-2.28)^b^	1.72 (1.30-2.29)^b^	1.45 (1.11-1.90)^f^	1.27 (0.98-1.65)^h^

Abbreviations: NO_2_, nitrogen dioxide; NO_x_, nitrogen oxides; OR, odds ratio; PM_2.5_, particulate matter with aerodynamic diameter of less than 2.5 μm; PM_10_, particulate matter with aerodynamic diameter of less than 10 μm.

^a^ Indicates association with the top quartile of the annualized mean of ambient air pollutants across the top 3 locations where participants spend their time. Includes participants with full data in model 6 (n = 1705). Analyses account for the nonindependence of twin observations.

^b^ *P* < .001.

^c^ *P* < .05.

^d^ Includes family socioeconomic status, family psychiatric history, and maternal psychosis.

^e^ Includes adolescent smoking, cannabis dependence, and alcohol dependence.

^f^ *P* < .01.

^g^ Includes neighborhood socioeconomic status, neighborhood crime rates, social cohesion, and neighborhood disorder.

^h^ *P* > .05 and P < .10.

### Sensitivity Analyses

We repeated analyses using urbanicity as an additional control variable to account comprehensively for urban factors correlated with air pollution. In short, main associations were not substantially changed after controlling for urbanicity (eTable 3 in the Supplement).

We repeated the analyses for participants who lived at the same address from 12 to 18 years of age (1472 [71.4%]). By restricting analyses in this way, we kept neighborhood conditions (including air pollution) as consistent over time as possible, with the caveat that neighborhood conditions tend to gradually change over time. In short, associations (from the regression and mediation models) were similar (albeit slightly stronger) for this subsample of adolescents who lived at the same address from 12 to 18 years of age (eTables 4 and 5 in the Supplement). For example, NO_2_ exposure (OR, 1.79; 95% CI, 1.28-2.50), NO_x_ exposure (OR, 1.76; 95% CI, 1.27-2.46), and PM_2.5_ exposure (OR, 1.60; 95% CI, 1.17-2.18) were significantly associated with adolescent psychotic experiences after considering all confounders.

We repeated the analyses using different thresholds for the pollution variables (full-scale continuous variables, dichotomized using WHO guidelines, and dichotomized at mean, and a 4-level variable). Odds for psychotic experiences were generally elevated among adolescents with higher vs lower pollution exposure, regardless of the threshold used (eTable 6 in the Supplement). For example, after considering all confounders and when air pollutants were dichotomized at the mean, the association of NO_2_ with adolescent psychotic experiences was an OR of 1.27 (95% CI, 0.99-1.62); for NO_x_, an OR of 1.32 (95% CI, 1.03-1.68); for PM_2.5_, an OR of 1.16 (95% CI, 0.92-1.47); and for PM_10_, an OR of 1.29 (95% CI, 1.02-1.63).

We repeated the analyses with clinically verified adolescent psychotic symptoms as the outcome. Effect sizes were similar to those found for adolescent psychotic experiences, although associations were not statistically significant after simultaneously adjusting for all potential confounders (eTable 7 in the Supplement). For example, after considering all confounders the association of NO_2_ with adolescent psychotic symptoms was an OR of 1.76 (95% CI, 0.82-3.79); for NO_x_, an OR of 1.79 (95% CI, 0.84-3.83), for PM_2.5_, an OR of 1.47 (95% CI, 0.68-3.14); and for PM_10_, an OR of 1.48 (95% CI, 0.69-3.14).

We also repeated the analyses using a 2-pollutant model (NO_x_ and PM_2.5_) to examine copollutant confounding. The associations arising from PM_2.5_ were null when simultaneously modeled with NO_x_. In contrast, the associations arising from NO_x_ were largely unchanged (eTable 8 in the Supplement). This finding suggests that associations arising from PM_2.5_ were driven by NO_x_/NO_2_ or another factor correlated with NO_x_/NO_2_.

### Does Air Pollution Explain the Association Between Urban Residency and Adolescent Psychotic Experiences?

As previously reported,^31^ psychotic experiences were significantly more common among adolescents residing in the most urban vs rural neighborhoods at 18 years of age (OR, 1.93; 95% CI, 1.35-2.75). Table 3 displays mediation models of the association between the most urban residency and adolescent psychotic experiences, split into the direct pathway (the part of the association not explained by the specified air pollutant, plus measurement error) and indirect pathway (the part of the association that is statistically mediated via the specified air pollutant). Mediation model 1 shows that NO_2_ (45%; OR, 1.34; 95% CI, 1.11-1.61) and NO_x_ (45%; OR, 1.34; 95% CI, 1.12-1.61) each significantly mediated (significant indirect ORs) the association between urbanicity and adolescent psychotic experiences. After considering potential confounders (mediation model 2), the mediatory pathways of NO_2_ (OR, 1.25; 95% CI, 1.07-1.45) and NO_x_ (OR, 1.26; 95% CI, 1.08-1.47) remained statistically significant, with each explaining 55% and 58% of the association between urban residency and adolescent psychotic experiences, respectively. Nitrogen dioxide and NO_x_ were of course highly correlated (*r* = 0.93; *P* < .001). When NO_2_ and NO_x_ were simultaneously entered as mediators, together they statistically explained 60% of the adjusted association between most urban residency and adolescent psychotic experiences. Thus, mediatory pathways via NO_2_ and NO_x_ largely overlapped and cannot be disentangled in this study. Mediation analyses were conducted using the 3-level urbanicity variable. Mediatory pathways arising for the intermediate urban settings are shown in eTable 9 in the Supplement.

**Table 3.** Mediation Model of the Association Between Most Urban Residency and Adolescent Psychotic Experiences via Air Pollutant Exposure^a^

Air Pollutant Mediator	Mediation Model 1^b^				Mediation Model 2^c^			
	OR (95% CI)			% Mediated	OR (95% CI)			% Mediated
	Total	Direct	Indirect		Total	Direct	Indirect	
NO_2_	1.91 (1.34-2.73)^d^	1.43 (0.96-2.12)^e^	1.34 (1.11-1.61)^f^	45^g^	1.49 (0.94-2.36)^e^	1.19 (0.75-1.91)	1.25 (1.07-1.45)^f^	55^g^
NO_x_	1.91 (1.34-2.72)^d^	1.43 (0.96-2.12)^e^	1.34 (1.12-1.61)^f^	45^g^	1.49 (0.94-2.36)^e^	1.18 (0.74-1.89)	1.26 (1.08-1.47)^f^	58^g^
PM_2.5_	1.93 (1.35-2.75)^d^	1.66 (1.13-2.43)^h^	1.16 (1.00-1.35)^e^	23	1.50 (0.95-2.37)^e^	1.35 (0.85-2.15)	1.11 (0.99-1.23)^e^	25
PM_10_	1.92 (1.35-2.74)^d^	1.81 (1.26-2.60)^f^	1.06 (0.99-1.14)^e^	9	1.50 (0.95-2.37)^e^	1.47 (0.93-2.32)	1.02 (0.99-1.06)	5

Abbreviations: NO_2_, nitrogen dioxide; NO_x_, nitrogen oxides; OR, odds ratio; PM_2.5_, particulate matter with aerodynamic diameter of less than 2.5 μm; PM_10_, particulate matter with aerodynamic diameter of less than 10 μm.

^a^ Mediation models were calculated separately for each air pollutant. This explains the very small differences in total ORs between models. The final mediation model simultaneously estimated the mediatory effects of NO_2_ and NO_x_. Analyses included participants with full data in model 2 (n = 1705). Mediatory percentages are rounded to whole numbers. Note that mediation analyses were conducted using the 3-level urbanicity variable. Only the results for most urban settings are reported. Mediatory pathways arising for the intermediate urban settings are shown in eTable 9 in the Supplement. Analyses account for the nonindependence of twin observations.

^b^ Indicates the unadjusted association between most urban (vs rural) residency at 18 years of age and adolescent psychotic experiences, split into the total effects (overall association between urbanicity and adolescent psychotic experiences), direct effects (the part of the association that is not explained by mediators in the model, plus measurement error), and the indirect effects (the part of the association that is statistically mediated via specified pollutants in the model).

^c^ Indicates total, direct, and indirect effects of most urban residency on adolescent psychotic experiences, adjusted simultaneously for family factors, childhood psychotic symptoms, adolescence substance use, and neighborhood factors.

^d^ *P* < .001.

^e^ *P* > .05 and *P* < .10.

^f^ *P* < .01.

^g^ Indicates significant indirect (mediation) pathways at *P* <.05.

^h^ *P* < .05.

## Discussion

In this study, adolescents exposed to high levels of outdoor air pollution were more likely to report psychotic experiences. Associations were not explained by a range of potential individual-, family-, and neighborhood-level confounders. Levels of NO_2_ and NO_x_ statistically explained 60% of the association between urban residency and adolescent psychotic experiences.

Several mechanisms might explain the association between air pollution and adolescent psychotic experiences. Air pollutants have potent oxidative effects on lipids and proteins.^14^ Biopsy and postmortem studies of children and adolescents have linked air pollution with disruption of the nasal epithelium and blood-brain barrier, as well as neuroinflammation and neurodegeneration in regions including the frontal cortex and olfactory bulb.^59,60^ Although the etiology of psychotic experiences remains equivocal, subtle abnormalities in brain structure and function have been identified, such as neuroinflammatory markers^61^ and aberrant prefrontal activity.^62,63^ Thus, air pollution could increase the risk for psychotic experiences by directly influencing the brain. Such influences are likely to be cumulative. However, in vitro rodent studies have demonstrated widespread neuroinflammation and neurotoxic effects, after even short-term exposure to air pollutants.^64,65^ In addition, higher developmental exposure to air pollution has been linked to lower serum vitamin D levels (potentially through reduced sunlight exposure),^66,67^ which have in turn been associated with increased risk for childhood psychotic experiences.^68^ The association among air pollution, vitamin D, and psychotic experiences warrants research. Furthermore, NO_2_ and NO_x_ are strongly linked to vehicle emissions.^11^ Findings therefore implicate road traffic, and by extension, noise pollution. Noise pollution has been linked to stress,^69^ sleep disturbance,^70^ and cognitive impairments among children and adolescents,^71^ which have in turn been associated with subclinical psychotic phenomena.^72,73,74^ Therefore, the association of NO_2_ and NO_x_ with adolescent psychotic experiences may have been linked more generally to road traffic and noise pollution experienced by participants living near busy roads.

### Future Directions

This study demonstrates the feasibility and value of linking high-resolution data on air pollution with rich phenotypic data. Our findings require replication. Further research is needed in this and other cohorts to explore the association of early-life exposure to air pollution with psychotic symptoms, psychotic disorders, and other psychiatric problems such as depression and anxiety to examine specificity. In addition, the mental health correlates of air pollution in low- and middle-income countries require attention. Air pollution levels (outdoor and household) in such countries can far exceed those in the West,^75,76^ with approximately 50% of the world’s population (predominantly in developing countries) relying on indoor combustion of coal and biomass for domestic energy.^76^ Paradoxically, recent research suggests that the urbanicity-psychosis association is a Western phenomenon, with null findings reported for low- and middle-income countries.^77^ One potential reason for this could be that air pollution (particularly household) follows less of an urbanicity gradient in developing countries.

### Implications

Pending replication, our findings have research, clinical, and public health implications. From a research perspective, findings highlight air pollution as another potential factor linking the urban environment to early psychotic phenomena. From a clinical perspective, a small but significant minority of youths who experience psychotic phenomena go on to develop clinical psychosis.^23^ Because early psychotic phenomena are also associated with numerous other adult psychiatric problems, our study provides further evidence implicating air pollution in adult psychosis and psychopathological disorders more broadly. From a public health perspective, the pollutants we have examined have legally binding limits set by the European Union.^10^ European levels of these air pollutants have slowly declined in recent years.^12^ However, NO_2_ was significantly associated with adolescent psychotic experiences in our study, despite the threshold being lower than international guidelines. European and global targets for air pollution may thus be too lax.

### Strengths and Limitations

To our knowledge, this study is the first to explore the association between air pollution and adolescent psychotic experiences. The air pollution measures achieve high geographic resolution for mental health research and demonstrate good model performance; thus, we can be reasonably sure that the measures closely represent the adolescents’ true ambient exposure. In addition, we have incorporated pollution data on 3 locations where participants spent their time, providing a comprehensive picture of exposure. We were also able to control for a range of individual-, family-, and neighborhood-level factors that might confound the association.

Several limitations should also be considered. First, our measure of adolescent psychotic experiences was not clinically verified. However, point estimates for clinically verified psychotic symptoms were similar (although nonsignificant) to those found for adolescent psychotic experiences, suggesting that air pollutants might be etiologically relevant across the psychosis continuum. Second, pollution was modeled for the year leading up to the interviews at 18 years of age. As such, we were not able to examine associations of early-life or cumulative exposure to air pollution with psychotic experiences. Modeling pollution data for earlier childhood addresses in this cohort will be important. However, children tend to live in consistent neighborhood settings throughout childhood and adolescence. This feature of neighborhood research makes it difficult to differentiate timing from duration. Sensitivity analyses for adolescents who did not move between residences from 12 to 18 years of age (and therefore certainly lived in consistent neighborhood conditions throughout adolescence) nevertheless supported the validity of the findings. Third, emerging findings suggest that urban residents carry a greater burden of genetic risk for schizophrenia.^78^ However, associations were robust to adjustment for proxy measures of genetic risk, including family psychiatric history and maternal psychosis. Fourth, to capture the worst levels of pollution and create parity between measures, pollution variables were dichotomized at the highest quartile. Dichotomization loses information, but sensitivity analyses using alternative variable thresholds supported the main findings. Fifth, air pollutants were all highly correlated, introducing copollutant confounding (as demonstrated in eTable 8 in the Supplement) and preventing us from disentangling the associations of NO_2_ and NO_x_. Furthermore, NO_2_ and NO_x_ could also be markers of other air pollutants not examined in our study.^79^ Finally, the E-Risk cohort is a twin sample, and findings might not generalize to singletons. However, the E-Risk cohort is representative of the UK population for key sociodemographic indices used in this study.

## Conclusions

In this study, youths exposed to the highest levels of air pollution were more likely to report psychotic experiences. In a rapidly urbanizing world, global efforts are needed to reduce air pollution levels and protect the mental (as well as physical) health of young urban citizens.

## Notes

Supplement. **eMethods.** Sample and Measures **eTable 1. **Types of Locations That Participants Reported Spending Most of Their Time at Age 18 **eTable 2. **Performance Statistics of CMAQ-Urban for 2012 **eTable 3. **Sensitivity Analyses Using Urbanicity as an Additional Control Variable **eTable 4. **Sensitivity Analyses for Nonmovers With Model Adjustment **eTable 5. **Sensitivity Analysis for Nonmovers Mediation Model **eTable 6. **Sensitivity Analyses Using Difference Thresholds **eTable 7. **Sensitivity Analyses for Adolescent Psychotic Symptoms **eTable 8. **Sensitivity Analysis of 2-Pollutant Model of the Association Between Annualized Average Levels of NO_x_ and PM_2.5_ and Adolescent Psychotic Experiences **eTable 9. **Mediation Model of the Association Between Urban and Intermediate Residency and Adolescent Psychotic Experiences via Air Pollutants
