# Supplementary material for: Association of Air Pollution Exposure With Psychotic Experiences During Adolescence
Source: JAMA Psychiatry. 2019 Mar 27;76(6):614–23. doi: 10.1001/jamapsychiatry.2019.0056 (PMC6499472; doi:10.1001/jamapsychiatry.2019.0056)
Supplement: Supplement. — eMethods. Sample and Measures eTable 1. Types of Locations That Participants Reported Spending Most of Their Time at Age 18 eTable 2. Performance Statistics of CMAQ-Urban for 2012 eTable 3. Sensitivity Analyses Using Urbanicity as an Additional Control Variable eTable 4. Sensitivity Analyses for Nonmovers With Model Adjustment eTable 5. Sensitivity Analysis for Nonmovers Mediation Model eTable 6. Sensitivity Analyses Using Difference Thresholds eTable 7. Sensitivity Analyses for Adolescent Psychotic Symptoms eTable 8. Sensitivity Analysis of 2-Pollutant Model of the Association Between Annualized Average Levels of NOx and PM2.5 and Adolescent Psychotic Experiences eTable 9. Mediation Model of the Association Between Urban and Intermediate Residency and Adolescent Psychotic Experiences via Air Pollutants [file jamapsychiatry-76-614-s001.pdf]

Supplementary Online Content

Newbury JB, Arseneault L, Beevers S, et al. Association of air pollution exposure with psychotic experiences during adolescence. *JAMA Psychiatry*. Published online March 27, 2019. doi:10.1001/jamapsychiatry.2019.0056

eMethods. Sample and Measures

eTable 1. Types of Locations That Participants Reported Spending Most of Their Time at Age 18

eTable 2. Performance Statistics of CMAQ-Urban for 2012

eTable 3. Sensitivity Analyses Using Urbanicity as an Additional Control Variable

eTable 4. Sensitivity Analyses for Nonmovers With Model Adjustment

eTable 5. Sensitivity Analysis for Nonmovers Mediation Model

eTable 6. Sensitivity Analyses Using Difference Thresholds

eTable 7. Sensitivity Analyses for Adolescent Psychotic Symptoms

eTable 8. Sensitivity Analysis of 2-Pollutant Model of the Association Between Annualized Average Levels of NOx and PM2.5 and Adolescent Psychotic Experiences

eTable 9. Mediation Model of the Association Between Urban and Intermediate Residency and Adolescent Psychotic Experiences via Air Pollutants

This supplementary material has been provided by the authors to give readers additional information about their work.

© 2019 Newbury et al. *JAMA Psychiatry.*

eMethods. Sample and Measures

Supplementary Methods

Sample

Participants were members of the Environmental Risk (E-Risk) Longitudinal Twin Study, which investigates how genetic and environmental factors shape children's development. The sampling frame from which the E-Risk families were drawn was two consecutive birth cohorts (1994 and 1995) in a birth register of twins born in England and Wales.[1] Of the 15,906 twin pairs born in these two years, 71% joined the register. The E-Risk Study probability sample was drawn using a high-risk stratification strategy. High-risk families were those in which the mother had her first birth when she was 20 years of age or younger. This sampling frame was used (1) to replace high risk families who were selectively lost to the register via non-response and (2) to ensure sufficient base rates of environmental risk factors. Age at first childbearing was used as the risk-stratification variable because it was present for virtually all families in the register, it is relatively free of measurement error, and early childbearing is associated with a host of other difficulties and is a known risk factor for children's problem behaviours.[2,3] The high-risk sampling strategy resulted in a final sample in which one-third of Study mothers constitute a 160% oversample of mothers who were at high risk based on their young age at first birth (13–20 years), while the other two-thirds of Study mothers accurately represent all mothers in the general population (13–48 years) in England and Wales in 1994–95 (estimates derived from the General Household Survey[4]).

The Study sought a sample size of 1,100 families to allow for attrition in future years of the longitudinal study while retaining statistical power. An initial list of families who had same-sex twins was drawn from the register to target for home-visits, with a 10% oversample to allow for nonparticipation. Same-sex twin pairs were selected to simplify twin analyses. Of the 1,203 families from the initial list who were eligible for inclusion, 1,116 (93%) participated in home-visit assessments when the twins were age 5 years, forming the base sample for the study (2,232 children): 4% of families refused, and 3% were lost to tracing or could not be reached after many attempts. With parent's permission, questionnaires were posted to the children's teachers, and teachers returned questionnaires for 94% of cohort children. Zygosity was determined using a standard zygosity questionnaire, which has been shown to have 95% accuracy.[5] Ambiguous cases were zygosity-typed using DNA. Subsequently, all participants who provided a DNA sample at any point across the study phases (97%) have been genotyped and had their zygosity checked. The sample includes 56% monozygotic (MZ) and 44% dizygotic (DZ) twin pairs. Sex is evenly distributed within zygosity (49% male). All families are English speaking, and the majority (93.7%) are White.

Attrition has been minimal, and data has been successfully collected from 98% (at age 7 years), 96% (at age 10 years), 96% (at age 12 years), and most recently in 2012–2014, 93% of the original sample (at age 18 years). Home-visits at ages 5, 7, 10, and 12 years included face-to-face assessments with participants as well as their mother (or primary caregiver); the home-visit at age 18 included interviews only with the participants, and questionnaires completed by co-informants (caregivers and other family members). Each twin participant was assessed by a different interviewer. Most participants (71.4%; N=1475) lived at the same address between ages 12 and 18. In addition, adolescents who did move house tended to move to similar neighborhoods: 87.0% of movers who lived in urban/intermediate neighborhoods at age 12 also lived in urban/intermediate neighborhoods at age 18. The Joint South London and Maudsley and the Institute of Psychiatry Research Ethics Committee approved each phase of the study. Parents gave informed consent at ages 5–12. Participants gave assent at ages 5–12 and informed consent at age 18.

Measures

Childhood/adolescent psychotic phenomena. To measure childhood psychotic symptoms, E-Risk families were visited by mental health trainees or professionals when children were aged 12. Interviewers had no prior knowledge about the child. Different staff members interviewed the child's parents. Each child was privately interviewed about seven psychotic symptoms they may have experienced throughout childhood, with items pertaining to delusions and hallucinations including: Have other people ever read your thoughts? Have you ever

© 2019 Newbury et al. *JAMA Psychiatry.*

believed that you were being sent special messages through the television or radio, or that a programme has been arranged just for you alone? Have you ever thought you were being followed or spied on? Have you ever heard voices that other people cannot hear? Have you ever felt like you were under the control of some special power? Have you ever known what another person was thinking, like you could read their mind? Have you ever seen something or someone that other people could not see? The item choice was guided by the Dunedin Study's age-11 interview protocol[6] and an instrument prepared for the Avon Longitudinal Study of Parents and Children.[7] Interviewers coded each experience 0, 1, 2 indicating respectively "not present", "probably present", and "definitely present". A conservative approach was taken in designating a child's report as a symptom. First, the interviewer probed using standard prompts designed to discriminate between experiences that were plausible (e.g., "I was followed by a man after school") and potential symptoms (e.g., "I was followed by an angel who guards my spirit"), and wrote down the child's narrative description of the experience. Second, items and interviewer notes were assessed by a psychiatrist expert in schizophrenia, a psychologist expert in interviewing children, and a child and adolescent psychiatrist to verify the validity of the symptoms (but without consulting other data sources about the child or family). Third, because children were twins, experiences limited to the twin relationship (e.g., "My twin and I often know what each other are thinking") were coded as "not a symptom". Children were only designated as experiencing psychotic symptoms if they reported at least one definite, clinically-verified symptom. At age 12, 5.9% (N=125) of children reported experiencing psychotic symptoms (referred to as childhood psychotic symptoms).

The same items and clinical verification procedure was used when participants were interviewed at age 18, this time enquiring about psychotic symptoms they may have experienced since age 12. At age 18, 2.9% (N=59) of participants reported experiencing psychotic symptoms since age 12 that were clinically verified (referred to as adolescent psychotic symptoms). These rates are similar to those reported for community samples of children and adolescents in other studies using clinical verification procedures.[8,9] The comparatively low prevalence of psychotic symptoms at age 18 versus age 12 is also consistent with findings from other studies showing an attenuating rate of psychotic symptoms from childhood to adulthood.[10,11] Furthermore, psychotic symptoms in this cohort have previously been shown to have good construct validity, sharing many of the same genetic, social, neurodevelopmental, and behavioural risk factors and correlates as adult psychotic disorders.[12]

To obtain a broader measure of adolescent psychotic *experiences* during the age 18 interviews, participants were asked six items about unusual feelings and thoughts in addition to the seven hallucination/delusion items. These items drew on item pools since formalised in prodromal psychosis screening instruments including the Prevention through Risk Identification, Management and Education (PRIME)-screen[13] and the Structured Interview for Psychosis-Risk Syndromes (SIPS).[14] These additional items included: I have become more sensitive to lights or sounds; I feel as though I can't trust anyone; I worry that my food may be poisoned; People or places I know seem different; I believe I have special abilities or powers beyond my natural talents; My thinking is unusual or frightening. Interviewers coded each of the 13 items (7 original hallucination/delusion items plus 6 additional unusual experiences items) 0, 1, 2, indicating respectively "not present", "probably present" and "definitely present". Responses to each of the 13 items (none, probable, definite) were summed to create a psychotic experiences scale (potential range=0–26, actual range=0–18, M=1.19, SD=2.58). The psychotic experiences measure did not involve clinical verification, meaning that this is a self-report measure capturing a broader range of mild, moderate and potentially clinically pertinent hallucinations, delusions, and other unusual feelings and thoughts. Since there were low numbers of adolescents with high psychotic experiences scores (e.g., only 1.0% [N=21] of participants had a psychotic experiences score of 13 or more), scores were placed into an ordinal scale to tackle the skewed distribution while retaining more information than a binary score. Just over 30% of participants had at least one psychotic experience between ages 12 and 18: 69.8% reported no psychotic experiences (coded 0; N=1,440), 15.5% reported 1 or 2 psychotic experiences (coded 1; N=319), 8.1% reported 3–5 psychotic experiences (coded 2: N=166), and 6.7% reported 6 or more psychotic experiences (coded 3: N=138). This 30.2% prevalence is similar to the prevalence of self-reported psychotic experiences in other community samples of teenagers and young adults.[15-17]

Ambient air pollution. Pollution exposure estimates were linked to the latitude-longitude coordinates of participants' residential addresses (or where the participant spent most of their time) plus two additional addresses that the participants' reported spending their time in 2012, when the twins were aged 17 years. The most common locations were home, school, work, and shops, as described in the eTable 1. Pollution data for the primary addresses were available for 97.5% (N=2014) of the age-18 cohort (see eTable 1). Pollution estimates were modelled using CMAQ-urban, which is a coupled regional Chemical Transport model and street-scale

© 2019 Newbury et al. *JAMA Psychiatry.*

dispersion model. CMAQ-urban uses a new generation of road traffic emissions inventory in the UK to model air quality down to individual streets, providing hourly estimates of pollutants at 20x20 metre grid points throughout the UK (i.e., address-level). Full details on the creation and validation of this model have been described previously.[18,19] Participants' exposure to several pollutants has been estimated by averaging the levels of the pollutant across the year at up to three locations that participants reported spending most of their time in, and then averaging this across the locations (i.e., [annual pollution exposure in Location 1 + Location 2 + Location 3] ÷ 3). Data for the proportion of time spent at each location were not available for participants, therefore the pollution measures are simply averaged rather than weighted. Pollutants include NO_2 (nitrogen dioxide: regulated gaseous pollutant linked to road traffic and industrial activity), NO_x (nitrogen oxides: measure of road traffic and industrial activity composed of NO_2 and nitric oxide [NO]), and $PM_{2.5}$ and PM_{10} (particulate matter with an aerodynamic diameter <2.5 µm and <10µm, respectively: regulated pollutants composed of inorganic aerosols, carbonaceous aerosols and dusts). Annualized average pollution levels in the E-Risk cohort (in micrograms per cubic metre [$\mu g/m^3$]) were, for NO_2: M=20.18$\mu g/m^3$, SD=9.68, range=2.31-67.89$\mu g/m^3$; for NO_x: M=25.79$\mu g/m^3$, SD=16.28, range=2.48-151.08$\mu g/m^3$; for $PM_{2.5}$: M=11.24$\mu g/m^3$, SD=2.18, range=4.05-20.56$\mu g/m^3$; and for PM_{10}: M=16.40$\mu g/m^3$, SD=2.71, range=8.42-33.27$\mu g/m^3$. These levels are representative of the UK.[20] (See further statistical analysis of this dataset in the next section). In the E-Risk cohort, 4.5% (N=91) of participants were exposed to levels of NO_2 that exceeded WHO guidelines (40$\mu g/m^3$); 29.7% (N=598) of participants were exposed to NO_x levels that exceeded WHO guidelines (30$\mu g/m^3$); 80.8% (N=1,627) of participants were exposed to $PM_{2.5}$ levels that exceeded WHO guidelines (10$\mu g/m^3$); and 9.3% (N=187) of participants were exposed to PM_{10} levels that exceeded WHO guidelines (20$\mu g/m^3$). Since there were substantial differences between air pollutants in terms of the numbers of participants who exceeded the WHO thresholds, WHO cut-offs were not used in the main analyses. Instead, to capture the worst levels of air pollution and create parity between the measures, air pollutants were dichotomized at the top quartile of exposure for this sample (these quartile cut-offs were: NO_2=26.0 $\mu g/m^3$; NO_x=33.0$\mu g/m^3$; $PM_{2.5}$=12.4 $\mu g/m^3$; PM_{10}=17.6 $\mu g/m^3$). Though all air pollutants were highly correlated (all *r*'s=0.56-0.97, p's<0.001), we examined the associations of each pollutant with adolescent psychotic experiences in case of differential effects.

Evaluation of ambient air pollution. The modelled annual average concentrations of NO_2, NO_X, $PM_{2.5}$ and PM_{10} for 2012 were assessed prior to their use in the study. The predictions were compared against the ground-based measurements across Great Britain. The measurements were obtained from the Automatic Urban and Rural Network (AURN) and London Air Quality Network (LAQN) which include rural (16), urban background (81), roadside (49), kerbside (8), and industrial (4) sites. The performance statistics (see eTable 2) show good percentages of predictions within a factor of two of the measurements (FAC2 × 100), i.e., 94% for NO_2, 96% for NO_x, and 100% for $PM_{2.5}$ and PM_{10}. The model slightly underestimates NO_x (6.47 $\mu g/m^3$ or 8%) and $PM_{2.5}$ (0.41 $\mu g/m^3$ or 3%) while marginally overestimates NO_2 (1.14 $\mu g/m^3$ or 3%) and PM_{10} (0.46 $\mu g/m^3$ or 2%). The RMSE and r reveal that the spatial variations of the predicted NO_2 (RMSE=10.32 $\mu g/m^3$, r=0.90) and NO_x (RMSE=30.52 $\mu g/m^3$, r=0.89) are reasonably accurate although slightly less so for PM_{10} (RMSE=4.14 $\mu g/m^3$, r=0.77) and $PM_{2.5}$ (RMSE=2.80 $\mu g/m^3$, r=0.66). Further investigation has revealed that the prediction bias is largest at industrial and kerbside locations where emissions estimates are highly uncertain. Tackling uncertainties in emissions would further enhance the model performance.

Urbanicity. Urbanicity was derived from the ONS's Rural-Urban Definition for Small Area Geographies (RUC2011) classifications.[21] The ONS classifications utilised 2011 census data, and were designed for application to small geostatistical units (e.g. Output Areas). Detailed information on how the ONS created the RUC2011 classifications of urbanicity is available on the ONS webpages (https://www.gov.uk/government/uploads/system/uploads/attachment_data/file/239477/RUC11methodologypaperaug_28_Aug.pdf). Briefly, RUC2011 was created by laying a grid of hectare cells (100m^2) over England and Wales. Postcode addresses were assigned to cells, and residential densities were then calculated for increasing radii around each cell, providing each residential property with a density profile. This was combined with Output Area and contextual data, allowing each settlement to be assigned to one of ten urbanicity categories (Rural categories: sparse/non-sparse hamlets and isolated dwellings, sparse/non-sparse villages, sparse/non-sparse rural town and fringe; Urban categories: sparse/non-sparse city and town, and minor/major conurbations [conurbations are densely populated, large urban regions resulting from the expansion and coalescence of adjacent cities and towns]). ONS urbanicity scores were then assigned to every E-Risk family via the family's postcode when children were aged 5, 7, 10, 12 and 18. Given the low numbers within some rural categories, urbanicity was collapsed into three levels (1: "rural" = all rural categories [19.7% of participants at age 18]; 2:

© 2019 Newbury et al. *JAMA Psychiatry.*

“intermediate” = urban cities and towns [48.4% of participants at age 18]; and 3: “urban” = minor/major conurbations [31.9% of participants at age 18]). E-Risk participants are nationally-representative in terms of ONS urbanicity classifications; 31.9% of E-Risk participants lived in urban settings at age 18 compared to 36.1% nationwide; 48.4% versus 45.0% lived in intermediate settings; and 19.7% versus 18.9% lived in rural settings.

Family-level factors. Family socioeconomic status (SES) was measured via a composite of parental income, education, and occupation when participants were aged 5. The latent variable was categorized into tertiles (i.e., low–, medium–, and high–SES).[22] Family psychiatric history and maternal psychotic symptoms were both assessed when participants were aged 12. In private interviews, the mother reported on her own mental health history and the mental health history of her biological mother, father, sisters, brothers, as well as the twins' biological father.[23,24] This was converted to the proportion of family members with a history of any psychiatric disorder (coded 0–1.0; M=0.37, SD=0.27). For maternal psychotic symptoms, mothers were interviewed using the Diagnostic Interview Schedule (DIS)[25] for DSM-IV[26] which provides a symptom count for characteristic symptoms of schizophrenia (e.g. hallucinations, delusions, anhedonia): 16.6% of mothers had at least one symptom of schizophrenia.

Adolescent substance use. Adolescent tobacco smoking, cannabis dependence and alcohol dependence were assessed during face-to-face interviews at age 18 using the DIS.[25] Smoking status was determined based on whether the participant reported ever having been a daily smoker (yes/no); 26.2% (N=541) of participants met this criterion. Cannabis and alcohol dependence was determined based on DSM-IV criteria. At age 18, 4.3% (N=89) of participants met criteria for cannabis dependence and 12.8% (N=263) met criteria for alcohol dependence.

Neighborhood deprivation. Neighborhood-level deprivation was constructed using A Classification of Residential Neighborhoods (ACORN), a geodemographic discriminator developed by CACI Information Services (http://www.caci.co.uk/). Detailed information about ACORN's classification of neighborhood-level deprivation has been provided previously.[28-30] Briefly, CACI utilized over 400 variables from 2001 census data for Great Britain (e.g., educational qualifications, unemployment, housing tenure) and CACI's consumer lifestyle database. Following hierarchical-cluster-analysis, CACI created five distinct and homogeneous ordinal groups ranging from “Wealthy Achiever” (coded 1) to “Hard Pressed” (coded 5) neighborhoods. Neighborhood-level deprivation scores for the E-Risk families were then created by identifying the ACORN classification for that family's postcode when children were aged 18. E-Risk families are representative of UK households across the spectrum of neighborhood-level deprivation: 27.0% of E-Risk families live in “wealthy achiever” neighborhoods compared to 25.3% of households nation-wide; 7.2% vs 11.6% live in “urban prosperity” neighborhoods; 26.8% vs 26.9% live in “comfortably off” neighborhoods; 13.2% vs 13.9% live in “moderate means” neighborhoods; and 25.8% vs 20.7% live in “hard-pressed” neighborhoods.[45,46]

Neighborhood crime rates. Crime data in 2011 (the first year for which full street-level data was available), including information on the type of crime, date of occurrence, and approximate location, were accessed online as part of an open data sharing effort about crime and policing in England and Wales. Street-level crime data was extracted for each of the geospatial coordinates marking the family's home (for a full description see: https://data.police.uk/about/#location-anonymisation). Neighborhood crime rates were calculated by mapping a one-mile radius around each E-Risk Study participant's home and tallying the total number of crimes that occurred in the area each month (M=247, SD=274, range=1–1868). These monthly crime rates were calculated for 2011, and then collapsed into quartiles. This measure covers various forms of crime, including violent offenses (e.g., assaults), sexual offenses (e.g., rape), robberies, burglaries, theft, arson, and vandalism.

Neighborhood social conditions. Social conditions (i.e., social processes) were estimated via a postal survey sent in 2008 to residents living alongside E-Risk families when children were aged 12.[28,31] In Britain, a postcode area typically contains 15 households, with at most 100 households (e.g., large apartment block). This type of postcode-level resolution represents a marked advantage over many existing neighborhood studies in which much larger census tract or census block units of analysis are used. Our objective was to obtain multiple reporters (e.g., 2 or more) for each family's neighborhood (here defined to the street or apartment block level). Considering that the typical response rate for neighborhood surveys is approximately 30%,[32] questionnaires were sent to every household in the same postcode as the E-Risk families, excluding the E-Risk families themselves (addresses were identified from electoral roll records). The number of surveys sent per postcode ranged from 15 to 50 residences per neighborhood (M=18.96, SE=0.21). Excluding undelivered surveys (N=600), the overall response rate was 28.1% (5601/19926), similar to that previously found.[32] Survey

© 2019 Newbury et al. *JAMA Psychiatry.*

respondents typically lived on the same street or within the same apartment block as the children in our study. Surveys were returned by an average of 5.18 (SD=2.73) respondents per neighborhood (range=0–18 respondents). There were at least three responses for 80% of neighborhoods and at least two responses from 95% of the neighborhoods (N=5,601 respondents).[31] Most respondents had lived in the neighborhood for more than 5 years (83%), and only 1% of respondents had lived in the neighborhood for less than 1 year. In the present study, analyses control for social cohesion and neighborhood disorder. Social cohesion[33] (5 items, each coded 0-4) was assessed by asking residents whether their neighbors shared values and trusted and got along with each other, etc. Neighborhood disorder[34] (14 items, each coded 0-2) was assessed by asking residents whether certain problems affected their neighborhood, including muggings, assaults, vandalism, graffiti and deliberate damage to property, etc. Items within each neighborhood characteristic scale were averaged to create summary scores from each of the 5601 resident respondents. Neighborhood characteristic scores for each E-Risk family were then created by averaging the summary scores of respondents within that family's neighborhood. The resulting variables approach normal distribution across the full potential range (Social cohesion: M=2.23, SD=0.50, range=0-3.71; Neighborhood disorder: M=0.49, SD=0.34, range=0-1.93).

© 2019 Newbury et al. *JAMA Psychiatry.*

eTable 1. Types of Locations That Participants Reported Spending Most of Their Time at Age 18

**Location type**	**Top three locations**					
	Location 1		Location 2		Location 3	
	No.	%	No.	%	No.	%
Home	1876	93.15	2	0.11	-	-
School	55	2.73	907	47.86	2	0.15
Work	40	1.99	511	26.91	211	16.27
Shops	6	0.30	92	4.84	112	8.64
Other	37	1.84	387	20.38	972	74.94
Total	2,014	100	1,899	100	1,297	100

Note: Cumulative pollution exposure estimates were derived by averaging the pollution estimates at the three locations that participants reported spending most of their time. Not all participants provided a second (N=1,899) and third (N=1,297) address, therefore cumulative pollution exposure estimates incorporated one or two addresses for these participants.

© 2019 Newbury et al. *JAMA Psychiatry.*

eTable 2. Performance Statistics of CMAQ-Urban for 2012

Pollutant	Number of data	Observed mean (μg/m^3)	Modelled mean (μg/m^3)	FAC2	MB (μg/m^3)	NMB	RMSE (μg/m^3)	r
NO_2	109	37.75	38.90	0.96	1.14	0.03	10.32	0.90
NO_X	109	81.46	74.99	0.94	-6.47	-0.08	30.52	0.89
$PM_{2.5}$	86	13.03	12.62	1	-0.41	-0.03	2.80	0.66
PM_{10}	107	20.49	20.95	1	0.46	0.02	4.14	0.77

Note: FAC2, fraction of predictions within a factor of two; MB, mean bias; NMB, normalised mean bias; RMSE, root mean squared error; r, correlation coefficient.

© 2019 Newbury et al. *JAMA Psychiatry.*

eTable 3. Sensitivity Analyses Using Urbanicity as an Additional Control Variable

Model adjustment	Pollutants							
	NO_2		NO_x		$PM_{2.5}$		PM_{10}	
	OR	95% CI	OR	95% CI	OR	95% CI	OR	95% CI
Model 1 – Unadjusted	1.83***	1.42 – 2.36	1.84***	1.43 – 2.36	1.58***	1.23 – 2.03	1.39*	1.08 – 1.79
Model 2 – Urbanicity	1.58**	1.18 – 2.12	1.59**	1.19 – 2.12	1.32†	1.00 – 1.73	1.26†	0.97 – 1.63
Model 3 – All covariates simultaneously	1.63**	1.20 – 2.20	1.64**	1.22 – 2.22	1.34*	1.02 – 1.76	1.24	0.96 – 1.62

Indicates association between annualized mean levels of air pollutants and adolescent psychotic experiences. Note: CI, confidence interval; OR, odds ratio. *p<0.05 **p<0.01 ***p<0.001 † p>0.05 & <0.1 Model 1 – unadjusted association between air pollutants (annualized average of ambient air pollutants across top three locations that participants spent their time) and adolescent psychotic experiences. Model 2 – adjusted for urbanicity at age 18 (three-level variable: most urban, intermediate, rural). Model 3 – adjusted for all confounders simultaneously. Analyses conducted on participants with full data in Model 3: N=1,705. Analyses account for the non-independence of twin observations.

© 2019 Newbury et al. *JAMA Psychiatry.*

eTable 4. Sensitivity Analyses for Nonmovers With Model Adjustment

**Model adjustment**	**Pollutants**							
	NO_2		NO_x		$PM_{2.5}$		PM_{10}	
	OR	95% CI	OR	95% CI	OR	95% CI	OR	95% CI
Model 1 – Unadjusted	2.09***	1.57 – 2.80	2.06***	1.55 – 2.75	1.85***	1.39 – 2.45	1.40*	1.04 – 1.88
Model 2 – Family factors	2.06***	1.54 – 2.75	2.02***	1.52 – 2.69	1.82***	1.37 – 2.42	1.37*	1.02 – 1.84
Model 3 – Childhood psychotic symptoms	2.14***	1.60 – 2.86	2.10***	1.57 – 2.79	1.90***	1.43 – 2.53	1.37*	1.02 – 1.86
Model 4 – Adolescent substance use	2.02***	1.51 – 2.71	2.00***	1.50 – 2.67	1.79***	1.35 – 2.37	1.42*	1.06 – 1.91
Model 5 – Neighborhood factors	1.80**	1.29 – 2.52	1.79***	1.29 – 2.48	1.59**	1.17 – 2.17	1.20	0.88 – 1.64
Model 6 – All covariates simultaneously	1.79**	1.28 – 2.50	1.76**	1.27 – 2.46	1.60**	1.17 – 2.18	1.23	0.89 – 1.69

Indicates association between annualized average levels of air pollutants and adolescent psychotic experiences among participants who did not move house between ages 12 and 18

Note: CI, confidence interval; OR, odds ratio. *p<0.05 **p<0.01 ***p<0.001. Model 1 – unadjusted association between air pollutants (annualized average of ambient air pollutants across top three locations that participants spent their time) and adolescent psychotic experiences, for the 71.4% of participants who did not move house between ages 12 and 18. Model 2 – adjusted for family factors (family socioeconomic status, family psychiatric history, maternal psychosis). Model 3 – adjusted for childhood psychotic symptoms. Model 4 – adjusted for adolescent substance use (adolescent smoking, cannabis dependence, alcohol dependence). Model 5 – adjusted for neighborhood factors (neighborhood deprivation [index of multiple deprivation], neighborhood crime rates, social cohesion, neighborhood disorder). Model 6 – adjusted for all confounders simultaneously. Analyses conducted on non-movers, with full data in Model 6: N=1,289. Analyses account for the non-independence of twin observations.

© 2019 Newbury et al. *JAMA Psychiatry.*

eTable 5. Sensitivity Analysis for Nonmovers Mediation Model

Air pollutant mediator	Mediation model 1					Mediation model 2			
	Total OR[a] (95% CI)	Direct OR (95% CI)	Indirect OR (95% CI)	% Mediated		Total OR[a] (95% CI)	Direct OR (95% CI)	Indirect OR (95% CI)	% Mediated
NO_2	1.85** [1.24, 2.76]	1.29 [0.83, 2.02]	1.43** [1.16, 1.76]	**58**		1.34 [0.77, 2.33]	1.10 [0.63, 1.92]	1.22* [1.04, 1.43]	**68**
NO_x	1.85** [1.24, 2.76]	1.30 [0.83, 2.05]	1.42** [1.15, 1.75]	**57**		1.34 [0.77, 2.33]	1.10 [0.62, 1.91]	1.23* [1.04, 1.45]	**71**
$PM_{2.5}$	1.87** [1.25, 2.79]	1.49† [0.96, 2.31]	1.25* [1.05, 1.50]	**36**		1.35 [0.78, 2.34]	1.20 [0.69, 2.10]	1.12† [1.00, 1.26]	39
PM_{10}	1.88** [1.26, 2.80]	1.77** [1.18, 2.67]	1.06 [0.98, 1.15]	9		1.35 [0.78, 2.34]	1.34 [0.77, 2.32]	1.01 [0.98, 1.03	2

Mediation model of the association between urban residency at age 18 and adolescent psychotic experiences via air pollutants

Note: CI, confidence interval; OR, odds ratio. *p<0.05 **p<0.01 † p>0.05 & <0.1. Bold text denotes significant indirect (mediation) pathways at p<0.05. [a] Mediation models were calculated separately for each air pollutant. This explains the very small differences in Total ORs between models. Mediation model 1 – the unadjusted association between most urban (versus rural) residency at age 18 and adolescent psychotic experiences, split into the total effects (overall association between urbanicity and adolescent psychotic experiences), direct effects (the part of the association that is not explained by mediators in the model, plus measurement error), and the indirect effects (the part of the association that is mediated via specified pollutants in the model) for the 71.4% of participants who did not move house between ages 12 and 18. Mediation model 2 – total, direct, and indirect effects of urban residency on adolescent psychotic experiences, adjusted simultaneously for family factors, childhood psychotic symptoms, adolescence substance use and neighborhood factors. Analyses conducted on non-movers, with full data in Model 6: N=1,289. Mediatory percentages are rounded to whole numbers. Analyses account for the non-independence of twin observations.

© 2019 Newbury et al. *JAMA Psychiatry.*

eTable 6. Sensitivity Analyses Using Different Thresholds

**Variable thresholds**		**Model 1**							
		Pollutants							
		NO_2		NO_x		$PM_{2.5}$		PM_{10}	
		OR	95% CI	OR	95% CI	OR	95% CI	OR	95% CI
Full scale pollutants		1.02**	1.01 – 1.03	1.01**	1.00 – 1.02	1.04	0.97 – 1.11	1.05*	1.00 – 1.09
WHO thresholds[a]		1.46	0.82 – 2.59	1.74***	1.37 – 2.21	0.94	0.70 – 1.28	1.16	0.77 – 1.77
Dichotomised at mean		1.41**	1.12 – 1.77	1.42**	1.13 – 1.79	1.25†	0.99 – 1.57	1.43**	1.14 – 1.79
Dichotomised at highest quartile[b]		1.83***	1.42 – 2.36	1.84***	1.43 – 2.36	1.58***	1.23 – 2.03	1.39*	1.08 – 1.78
Four-level variable (quartiles):	1	(Reference)	-	(Reference)	-	(Reference)	-	(Reference)	-
	2	0.72*	0.52 – 0.99	0.67*	0.48 – 0.92	0.84	0.60 – 1.16	1.14	0.82 – 1.59
	3	0.89	0.64 – 1.23	0.87	0.62 – 1.21	0.89	0.64 – 1.24	1.41*	1.03 – 1.95
	4	1.58**	1.15 – 2.18	1.54**	1.12 – 2.10	1.43*	1.04 – 1.97	1.65**	1.20 – 2.27
		**Model 2**							
		Pollutants							
		NO_2		NO_x		$PM_{2.5}$		PM_{10}	
		OR	95% CI	OR	95% CI	OR	95% CI	OR	95% CI
Full scale pollutants		1.01	1.00 – 1.03	1.01	1.00 – 1.02	1.02	0.95 – 1.09	1.03	0.99 – 1.08
WHO thresholds[a]		1.16	0.59 – 2.27	1.61***	1.23 – 2.11	0.97	0.70 – 1.33	1.06	0.68 – 1.65
Dichotomised at mean		1.27†	0.99 – 1.62	1.32*	1.03 – 1.68	1.16	0.92 – 1.47	1.29*	1.02 – 1.63
Dichotomised at highest quartile[b]		1.71***	1.28 – 2.28	1.72***	1.30 – 2.29	1.45**	1.11 – 1.90	1.27†	0.98 – 1.65
Four-level variable (quartiles):	1	(Reference)	-	(Reference)	-	(Reference)	-	(Reference)	-
	2	0.70*	0.50 – 0.99	0.65*	0.46 – 0.91	0.92	0.65 – 1.29	1.24	0.88 – 1.74
	3	0.83	0.59 – 1.17	0.83	0.59 – 1.16	0.92	0.66 – 1.29	1.36†	0.97 – 1.89
	4	1.41†	0.98 – 2.03	1.38†	0.96 – 1.98	1.37†	0.97 – 1.92	1.54*	1.09 – 2.15

© 2019 Newbury et al. *JAMA Psychiatry.*

Note: CI, confidence interval; OR, odds ratio. *p<0.05 Association between annualized average levels of air pollutants and adolescent psychotic experiences

**p<0.01 ***p<0.001 † p>0.05 & <0.1. Model 1 – unadjusted association between air pollutants and adolescent psychotic experiences. Model 2 – adjusted simultaneously for potential individual-, family-, and neighborhood-level confounders. Analyses conducted on participants with full data in Model 2: N=1,705. Analyses account for the non-independence of twin observations. [a] WHO air pollution thresholds: NO_2 = 40 µg/m^3 annual mean (exceeded by 4.5% of participants); NO_x = 30 µg/m^3 annual mean (exceeded by 29.7% of participants); $PM_{2.5}$ = 20 µg/m^3 annual mean (exceeded by 80.1% of participants); PM_{10} = 10 µg/m^3 annual mean (exceeded by 9.3% participants). When interpreting the associations arising from pollutants dichotomised at the WHO thresholds, please note the large differences between pollutants in terms of the numbers of participants who exceed the WHO thresholds. [b] Variable threshold used in main analysis.

© 2019 Newbury et al. *JAMA Psychiatry.*

eTable 7. Sensitivity Analyses for Adolescent Psychotic Symptoms

Model adjustment	Pollutants							
	NO_2		NO_x		$PM_{2.5}$		PM_{10}	
	OR	95% CI	OR	95% CI	OR	95% CI	OR	95% CI
Model 1 – Unadjusted	1.99*	1.06 – 3.75	1.98*	1.06 – 3.73	1.60	0.84 – 3.08	1.73†	0.93 – 3.22
Model 2 – Family factors	1.93*	1.03 – 3.61	1.92*	1.02 – 3.60	1.57	0.82 – 3.01	1.65	0.88 – 3.07
Model 3 – Childhood psychotic symptoms	1.93*	1.02 – 3.65	1.97*	1.05 – 3.72	1.65	0.85 – 3.20	1.62	0.86 – 3.02
Model 4 – Adolescent substance use	1.94*	1.02 – 3.69	1.94*	1.02 – 3.67	1.54	0.80 – 2.99	1.70†	0.91 – 3.19
Model 5 – Neighborhood factors	1.91†	0.90 – 4.04	1.89†	0.91 – 3.95	1.47	0.72 – 3.00	1.61	0.80 – 3.25
Model 6 – All covariates simultaneously	1.76	0.82 – 3.79	1.79	0.84 – 3.83	1.47	0.68 – 3.14	1.48	0.69 – 3.14

Association between annualized average levels of air pollutants and adolescent psychotic symptoms

Note: CI, confidence interval; OR, odds ratio. *$p<0.05$ † $p>0.05$ & <0.1. Model 1 – unadjusted association between air pollutants (annualized average of ambient air pollutants across top three locations that participants spent their time) and adolescent psychotic symptoms. Model 2 – adjusted for family factors (family socioeconomic status, family psychiatric history, maternal psychosis). Model 3 – adjusted for childhood psychotic symptoms. Model 4 – adjusted for adolescent substance use (adolescent smoking, cannabis dependence, alcohol dependence). Model 5 – adjusted for neighborhood factors (neighborhood deprivation [index of multiple deprivation], neighborhood crime rates, social cohesion, neighborhood disorder). Model 6 – adjusted for all confounders simultaneously. Analyses conducted on participants with full data in Model 6: N=1,705. Analyses account for the non-independence of twin observations.

© 2019 Newbury et al. *JAMA Psychiatry.*

eTable 8. Sensitivity Analysis of 2-Pollutant Model of the Association Between Annualized Average Levels of NOx and PM2.5 and Adolescent Psychotic Experiences

**Model adjustment**	**Pollutants**			
	NO_x		$PM_{2.5}$	
	OR	95% CI	OR	95% CI
Model 1 – Unadjusted	1.90**	1.30 – 2.77	0.96	0.66 – 1.40
Model 2 – All covariates simultaneously	1.82**	1.22 – 2.71	0.93	0.63 – 1.37

Note: CI, confidence interval; OR, odds ratio. **p<0.01 Model 1 – association between air pollutants (annualized average of ambient air pollutants across top three locations that participants spent their time) and adolescent psychotic experiences, unadjusted for confounders but mutually adjusted for co-pollutants (that is, NO_x and $PM_{2.5}$ are simultaneously entered as covariates). Model 2 – adjusted for all individual-, family-, and neighborhood-level confounders simultaneously. Analyses conducted on participants with full data in Model 2: N=1,705. Analyses account for the non-independence of twin observations.

© 2019 Newbury et al. *JAMA Psychiatry.*

eTable 9. Mediation Model of the Association Between Urban and Intermediate Residency and Adolescent Psychotic Experiences via Air Pollutants

Level of urbanicity	Air pollutant mediator	Mediation model 1					Mediation model 2			
		Total OR[a] (95% CI)	Direct OR (95% CI)	Indirect OR (95% CI)	% Mediated		Total OR[a] (95% CI)	Direct OR (95% CI)	Indirect OR (95% CI)	% Mediated
Intermediate	NO_2	1.34† [0.96, 1.89]	1.32 [0.94, 1.85]	1.02 [0.98, 1.06]	7		1.08 [0.73, 1.62]	1.11 [0.74, 1.66]	0.97 [0.93, 1.02]	0
	NO_x	1.34† [0.96, 1.89]	1.31 [0.93, 1.84]	1.03 [0.99, 1.06]	9		1.08 [0.73, 1.62]	1.10 [0.74, 1.65]	0.98 [0.94, 1.03]	0
	$PM_{2.5}$	1.35† [0.96, 1.89]	1.33 [0.95, 1.87]	1.01 [0.99, 1.04]	5		1.09 [0.73, 1.62]	1.10 [0.74, 1.65]	0.98 [0.95, 1.01]	0
	PM_{10}	1.34† [0.96, 1.89]	1.30 [0.93, 1.83]	1.03 [0.99, 1.07]	11		1.08 [0.73, 1.62]	1.08 [0.72, 1.61]	1.00 [0.98, 1.03]	4
Urban	NO_2	1.91*** [1.34, 2.73]	1.43† [0.96, 2.12]	1.34** [1.11, 1.61]	**45**		1.49† [0.94, 2.36]	1.19 [0.75, 1.91]	1.25** [1.07, 1.45]	**55**
	NO_x	1.91*** [1.34, 2.72]	1.43† [0.96, 2.12]	1.34*** [1.12, 1.61]	**45**		1.49† [0.94, 2.36]	1.18 [0.74, 1.89]	1.26** [1.08, 1.47]	**58**
	$PM_{2.5}$	1.93*** [1.35, 2.75]	1.66* [1.13, 2.43]	1.16† [1.00, 1.35]	23		1.50† [0.95, 2.37]	1.35 [0.85, 2.15]	1.11† [0.99, 1.23]	25
	PM_{10}	1.92*** [1.35, 2.74]	1.81** [1.26, 2.60]	1.06† [0.99, 1.14]	9		1.50† [0.95, 2.37]	1.47 [0.93, 2.32]	1.02 [0.99, 1.06]	5

Note: CI, confidence interval; OR, odds ratio. *p<0.05 **p<0.01 ***p<0.001 † p>0.05 & <0.1. Bold text denotes significant indirect (mediation) pathways at p<0.05. [a] Mediation models were calculated separately for each air pollutant. This explains the very small differences in Total ORs between models. The final mediation model simultaneously estimated the mediatory effects of NO_2 and NO_x (see Main Results). Mediation model 1 – the unadjusted association between intermediate/most urban (versus rural) residency at age 18 and adolescent psychotic experiences, split into the total effects (overall association between urbanicity and adolescent psychotic experiences), direct effects (the part of the association that is not explained by mediators in the model, plus measurement error), and the indirect effects (the part of the association that is statistically mediated via specified pollutants in the model). Mediation model 2 – total, direct, and indirect effects of intermediate/urban residency on adolescent psychotic experiences, adjusted simultaneously for family factors, childhood psychotic symptoms, adolescence substance use and neighborhood factors. Analyses conducted on participants with full data in Model 2: N=1,705. Mediatory percentages are rounded to whole numbers). Analyses account for the non-independence of twin observations.

© 2019 Newbury et al. *JAMA Psychiatry.*

References from Supplementary Materials

1. Trouton A, Spinath FM, Plomin R. Twins early development study (TEDS): A multivariate, longitudinal genetic investigation of language, cognition and behavior problems in childhood. *Twin Res.* 2002; 5(5): 444-448.
2. Maynard RA. *Kids having kids: Economic costs & social consequences of teen pregnancy*. Washington, DC: The Urban Insitute; 1997.
3. Moffitt TE, The E-Risk Study Team. Teen-aged mothers in contemporary Britain. *J Child Psychol Psychiatry.* 2002; 43(6): 727-742.
4. Bennett N, Jarvis L, Rowlands O, Singleton N, Haselden L. *Living in Britain: Results from the 1994 general household survey*. London: HMSO; 1996.
5. Price TS, Freeman B, Craig I, et al. Infant zygosity can be assigned by parental report questionnaire data. *Twin Res Hum Genet.* 2000; 3(3): 129-133.
6. Poulton R, Caspi A, Moffitt TE, et al. Children's self-reported psychotic symptoms and adult schizophreniform disorder: A 15-year longitudinal study. *Arch Gen Psychiatry.* 2000; 57(11): 1053-1058.
7. Schreier A, Wolke D, Thomas K, et al. Prospective study of peer victimization in childhood and psychotic symptoms in a nonclinical population at age 12 years. *Arch Gen Psychiatry.* 2009; 66(5): 527-536.
8. Dhossche D, Ferdinand R, van der Ende J, Hofstra M, Verhulst F. Diagnostic outcome of self-reported hallucinations in a community sample of adolescents. *Psychol Med.* 2002; 32(4): 619-627.
9. Horwood J, Salvi G, Thomas K, et al. IQ and non-clinical psychotic symptoms in 12-year-olds: Results from the ALSPAC birth cohort. *Br J Psychiatry.* 2008; 193(3): 185-191.
10. Kelleher I, Keeley H, Corcoran P, et al. Clinicopathological significance of psychotic experiences in non-psychotic young people: Evidence from four population-based studies. *Br J Psychiatry.* 2012; 201(1): 26-32.
11. Zammit S, Kounali D, Cannon M, et al. Psychotic experiences and psychotic disorders at age 18 in relation to psychotic experiences at age 12 in a longitudinal population-based cohort study. *Am J Psychiatry.* 2013; 170(7): 742-750.
12. Polanczyk G, Moffitt TE, Arseneault L, et al. Etiological and clinical features of childhood psychotic symptoms: Results from a birth cohort. *Arch Gen Psychiatry.* 2010; 67(4): 328-338.
13. Miller TJ, Cicchetti D, Markovich PJ, McGlashan TH, Woods SW. The SIPS-Screen: a brief self-report screen to detect the schizophrenia prodrome. *Schizophr Res.* 2004; 70 (suppl1): 78.
14. Miller TJ, McGlashan TH, Rosen JL, et al. Prodromal assessment with the structured interview for prodromal syndromes and the scale of prodromal symptoms: predictive validity, interrater reliability, and training to reliability. *Schizophr Bull.* 2003; 29(4): 703.
15. Spauwen J, Krabbendam L, Lieb R, Wittchen HU, van Os J. Does urbanicity shift the population expression of psychosis? *J Psychiatr Res.* 2004; 38(6): 613-618.
16. Yoshizumi T, Murase S, Honjo S, Kaneko H, Murakami T. Hallucinatory experiences in a community sample of Japanese children. *J Am Acad Child Adolesc Psychiatry.* 2004; 43(8): 1030-1036.
17. Yung AR, Nelson B, Baker K, et al. Psychotic-like experiences in a community sample of adolescents: Implications for the continuum model of psychosis and prediction of schizophrenia. *Aust N Z J Psychiatry.* 2009; 43(2): 118-128.
18. Beevers SD, Kitwiroon N, Williams ML, Carslaw DC. One way coupling of CMAQ and a road source dispersion model for fine scale air pollution predictions. *Atmos Environ.* 2012; 59: 47-58.
19. Carslaw DC. Defra urban model evaluation analysis - Phase 1. *Report to Defra, Environmental Research Group*. https://uk-air.defra.gov.uk/library/reports?report_id=654. Published 2011. Accessed August, 2018.
20. European Environment Agency. Air quality in Europe - 2017 report. https://www.eea.europa.eu/publications/air-quality-in-europe-2017. Published 2017. Accessed August, 2018.
21. Office for National Statistics. Urban and rural area definitions for policy purposes in England and Wales: Methodology (v1.0). https://assets.publishing.service.gov.uk/government/uploads/system/uploads/attachment_data/file/239477/RUC11methodologypaperaug_28_Aug.pdf. Published 2013. Accessed August, 2018.

© 2019 Newbury et al. *JAMA Psychiatry.*

22. Trzesniewski KH, Moffitt TE, Caspi A, Taylor A, Maughan B. Revisiting the association between reading achievement and antisocial behavior: New evidence of an environmental explanation from a twin study. *Child Dev.* 2006; 77(1): 72-88.

23. Milne B, Moffitt T, Crump R, et al. How should we construct psychiatric family history scores? A comparison of alternative approaches from the Dunedin Family Health History Study. *Psychol Med.* 2008; 38(12): 1793-1802.

24. Weissman MM, Wickramaratne P, Adams P, et al. Brief screening for family psychiatric history: The family history screen. *Arch Gen Psychiatry.* 2000; 57(7): 675-682.

25. Robins L, Cottler L, Bucholz K, Compton W. *Diagnostic Interview Schedule for DSM-IV (DIS-IV)*. St Louis, MO: Washington University School of Medicine; 1995.

26. American Psychiatric Association. *Diagnostic and Statistical Manual of Mental Disorders*. Washington, DC: American Psychiatric Association; 1994.

27. CACI Information Services. *ACORN user guide*. London, UK: CACI; 2006.

28. Odgers CL, Moffitt TE, Tach LM, et al. The protective effects of neighborhood collective efficacy on British children growing up in deprivation: A developmental analysis. *Dev Psychol.* 2009; 45(4): 942-957.

29. Odgers CL, Caspi A, Russell MA, et al. Supportive parenting mediates neighborhood socioeconomic disparities in children's antisocial behavior from ages 5 to 12. *Dev Psychopathol.* 2012; 24(3): 705-721.

30. Caspi A, Taylor A, Moffitt TE, Plomin R. Neighborhood deprivation affects children's mental health: Environmental risks identified in a genetic design. *Psychol Sci.* 2000; 11(4): 338-342.

31. Odgers CL, Caspi A, Bates CJ, Sampson RJ, Moffitt TE. Systematic social observation of children's neighborhoods using Google Street View: A reliable and cost-effective method. *J Child Psychol Psychiatry.* 2012; 53(10): 1009-1017.

32. Messner SF, Baumer EP, Rosenfeld R. Dimensions of social capital and rates of criminal homicide. *Am Sociol Rev.* 2004; 69(6): 882-903.

33. Sampson RJ, Raudenbush SW, Earls F. Neighborhoods and violent crime: A multilevel study of collective efficacy. *Science.* 1997; 277(5328): 918-924.

34. Sampson RJ, Raudenbush SW. Systematic social observation of public spaces: A new look at disorder in urban neighborhoods. *Am J Sociol.* 1999; 105(3): 603-651.

© 2019 Newbury et al. *JAMA Psychiatry.*
